# Population structure, genetic variability, and gene flow of the bean leaf beetle, *Cerotoma trifurcata* , in the Midwestern United States

**DOI:** 10.1093/jis/14.1.62

**Published:** 2014-01-01

**Authors:** Bamphitlhi Tiroesele, Steven R. Skoda, Thomas E. Hunt, Donald J. Lee, Jaime Molina-Ochoa, John E. Foster

**Affiliations:** 1 Department of Entomology, University of Nebraska-Lincoln, Lincoln, NE 68583-0816; 2 USDA-ARS-KBUSLIRL Screwworm Research Unit, Kerrville, TX 78028; 3 Department of Entomology, University of Nebraska Haskell Agricultural Laboratory, Concord, NE, 68728-2828; 4 Department of Agronomy and Horticulture, University of Nebraska-Lincoln, Lincoln, NE 68583-0915; 5 Coordinación General de Investigación Científica, Centro Universitario de Investigación y Desarrollo Agropecuario, Tecomán Colima 28930, México

**Keywords:** AFLP, genetic distance, geographic distance, soybean pests

## Abstract

Bean leaf beetle, *Cerotoma trifurcata* (Forster) (Coleoptera: Chrysomelidae), is a common pest of soybean in the Midwest United States. However, there are currently no reports on the genetic variability of *C. trifurcata* . This study examined 15–30 individuals from 25 sample locations to estimate genetic variability and gene flow within and among *C. trifurcata* from across the Mid- west. Amplified fragment length polymorphism generated 175 markers for analyses. Results from analysis of molecular variance (AMOVA) indicated that the majority of genetic variation was from within samples; only a small amount of the total variation was attributed to the variation among the samples. The GST for the entire *C. trifurcata* population indicated that the majority of genetic variation was found within the samples, further supporting the AMOVA results. The estimated average gene flow among the *C. trifurcata* samples was 1.83. The Mantel test revealed no indication of correlation between geographical and genetic distance for all the *C. trifurcata* samples. These findings show that *C. trifurcata* in the Midwest are genetically heterogeneous and part of a large, interbreeding population.

## Introduction

Bean leaf beetle, *Cerotoma trifurcata* (Forster) (Coleoptera: Chrysomelidae), is a pest of leguminous crops, especially soybean, in the United States. *Cerotoma trifurcata* adults directly damage the soybeans by feeding on the leaves, stems, and pods ( Smelser and Pedigo 1992 ), while the larvae feed on the plant root system ( Lundgren and Riedell 2008 ). It also causes indirect damage by transmitting soybean diseases such as bean pod mottle virus ( Hopkins and Muller 1984 ), soybean mosaic virus, yellow cowpea mosaic virus ( Jansen and Staples 1971 ), cowpea chlorotic mottle virus ( Walters and Dodds 1969 ), and southern bean mosaic virus ( Walters 1964 ). These viruses, especially bean pod mottle virus, reduce soybean yield ( Horn et al. 1973 ) and grain quality ( Hill et al. 2007 ). *Cerotoma trifurcata* overwinter as adults under leaf litter in wooden areas; the following spring, these overwintering individuals then attack the soybeans as they emerge.

Understanding the genetic background of insect pests can aid in understanding their evolution in changing environments, hence aiding in effecting their management in an agricultural ecosystem. Genetic fragmentation affects gene flow within several insect species ( Liebherr 1988 ; Crouau-Roy 1989 ). Sometimes distance alone can function as a barrier to genetic exchange among samples (Gonza- lez-Rodriguez et al. 2000; Ruggiero et al. 2004 ). Isolation caused by geographic barriers, habitat suitability, or distance is capable of restricting gene flow within the *C. trifurcata* population and could result in population fragmentations and genetic differentiation. It is important to characterize the genetic variability, gene flow, and ecological features of pest target populations prior to a large investment in large-scale efforts aimed at controlling insect pests ( Sluss and Graham 1979 ; Martinelli et al. 2007 ). The inability to detect or improper detection of differences between samples can lead to drastic and costly consequences in pest management. There are currently no studies on the genetic variability of *C. trifurcata* .

The advent of molecular genetic tools allowed extensive descriptions and analyses of insects ( Reineke et al. 1998 ), including *C. trifurcata* . Amplified fragment length polymorphism (AFLP) is a widely used, powerful technique for DNA profiling mostly used in assessing diversity within and among organisms that have varying genomic structures. Although AFLP produces dominant markers, it can be used to detect molecular genetic variations in DNA of any source or complexity without prior sequence knowledge ( Vos et al. 1995 ). The capability of generating a large number of polymorphic loci genome-wide, the high level of reproducibility, the quick start-up time, and the relatively low cost make it a very useful tool that overcomes the issue of dominant markers ( Savelkoul et al. 1999 ; Gerber et al. 2000 ). This study was of the genetic variability and gene flow of *C. trifurcata* specimens from the Midwest United States using AFLP.

## Materials and Methods

### Insect collection

**Figure 1.**
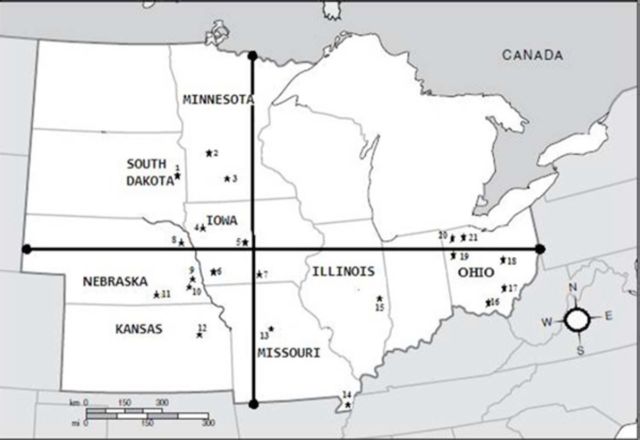
Sampling sites of Midwest (USA) *Cerotoma trifurcata* subpopulations used in this study. *1-Brookings, 2-Becker, 3- Lamberton, 4-Sutherland, 5-Ames, 6-Lewis, 7-Chariton, 8-Concord, 9-Ithaca (Mead), 10-Lincoln, 11-Clay Center, 12-Manhattan, 13- Columbia, 14-Pemiscot, 15-Savoy, 16-Logan, 17-Waverly, 18-Richland, 19-Hancock, 20-Hoytville, 21-Pemberville. High quality figures are available online.

Collection sites were divided into five regions (Central, South, West, East, and North) along two transects in the Midwest United States **(**Figure 1**)** . A total of 25 samples were taken. All samples were collected from soybean fields by using sweep nets ( Table 1 ). Two perpendicular transects were designed running from Minnesota/South Dakota to Missouri/Kansas, and from Nebraska to Illinois/Ohio. Iowa was regarded as the central area for the two transects. The samples were collected from an area spanning about 960 miles (west to east: 1545 km) by about 790 miles (north to south: 1,271 km). The Missis- sippi River separated East samples from the others, the Missouri River separated the Center from West samples, and North from South samples were quite distant. The West samples were collected from Lincoln, Mead, Concord, and Clay Center areas of Nebraska in 2008 and 2010 ( Table 1 ). The East samples were collected in 2003 (Waverly, Pemberville, and Hoytville in Ohio) and 2010 (Savoy, IL, and Logan, Richland, and Hancock in Ohio) ( Table 1 ). All other samples were collected in 2010 ( Table 1 ). The number of insects collected and used per location (sample) varied between 15 to more than 30. The collected *C. trifurcata* adults were stored in 95% alcohol until the samples reached the laboratory. The alcohol was then changed twice to avoid alcohol dilution by fluids from sampled adults, which can lead to DNA degradation, and then kept in the freezer at -80°C until processing for DNA isolation.

**Table 1.**
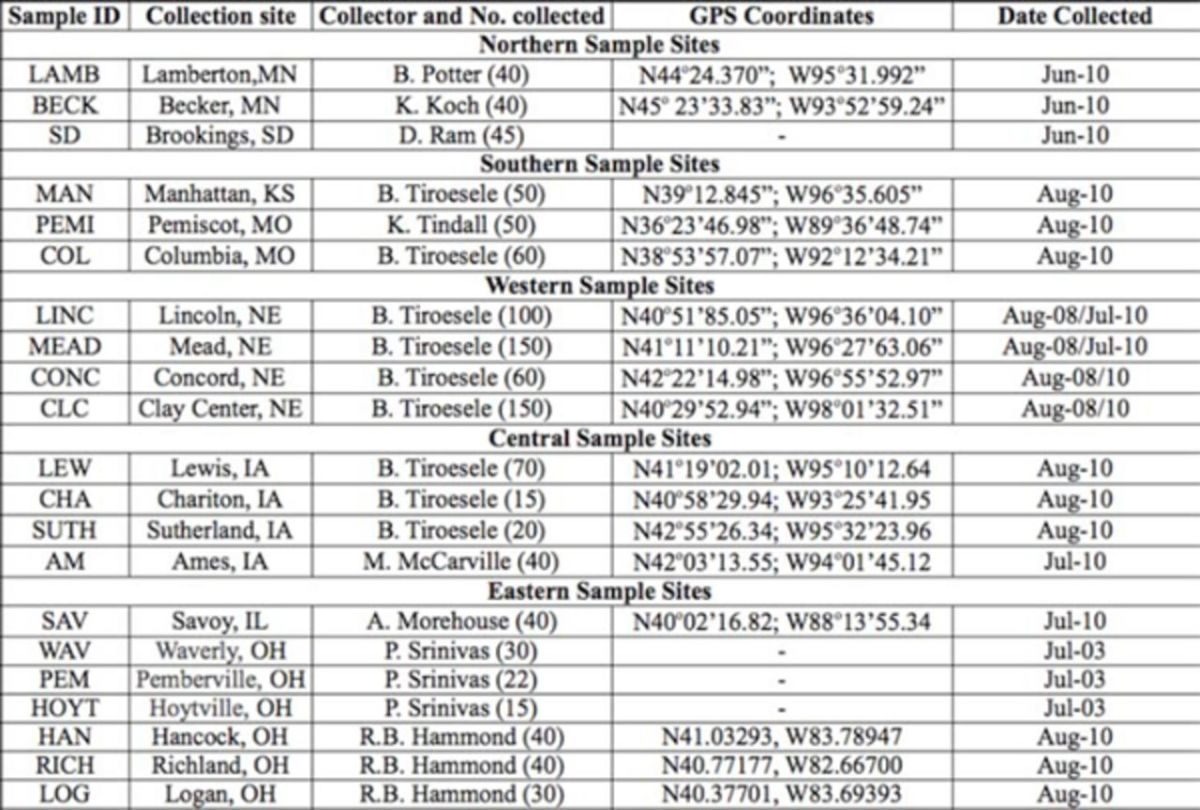
Code, collection site, collector, and date of collection of adult samples of *Cerotoma trifurcata* .

### DNA extraction and quantification

DNA was extracted from the thorax of *C. trifurcata* adults using a hexadecyltrimethylammoniumbromide (CTAB; Sigma-Aldrich, www.sigmaaldrich.com ) extraction protocol as modified by Clark (2005) . The frozen adults were first soaked and washed in a beaker of double distilled autoclaved water for 10 min. The adults were then prepared for DNA extraction by removing the gut, abdomen, and head, leaving only the thorax to use in the study. The thorax was homogenized in 500 µL of CTAB extraction buffer (100 mM Tris-HCL, 1.4 M NaCl, 0.02 M EDTA, 2% CTAB, and 0.2% β -mercapto ethanol) (Sig- ma-Aldrich). 10 µL Proteinase K (concentration of 200 µg/mL extraction buffer; Sigma-Aldrich) was added to the homogenate in each tube and then incubated for 1.5 hr at 65°C. RNase A (15 µL; 500 µg/mL concentration; Sigma-Aldrich) was added to the homogenates, and this was incubated for 2 hr at 37°C. After RNA and protein were removed from each sample, the homogenate was centrifuged at 14,000 rpm for 5 min at room temperature. The supernatant was then removed and placed in clean, 1.5-µL autoclaved microcentrifuge tubes. This supernatant was further extracted with 500 µL of chloroform:isoamyl alcohol (24:1) (Sigma-Aldrich) by centrifugation at 14,000 rpm for 20 min to separate the phases. The top, aqueous phase was transferred into a clean, autoclaved, 1.5-mL microcentrifuge tube, and the chloroform:isoamyl step was repeated. The aqueous phase was once again collected into another clean, autoclaved, 1.5-mL microcentrifuge tube. DNA was then precipitated by adding 400 µL chilled (-20°C) isopropanol to the aqueous phase and incubated at 4°C for at least 8 hr. After incubation, the precipitate was centrifuged at 12,000 rpm at 4°C for 30 min. The isopropanol was decanted; the DNA pellet was then rinsed with 500 µL 100% chilled ethanol (ETOH) and centrifuged at 12,000 rpm at 4°C for 5 min. The supernatant was poured off, the pellet was rinsed with 500 µL of 70% cold ETOH, and this was centrifuged for 5 min. The ETOH was decanted, and the pellet was then air dried at room temperature (24°C) for 50 min under the hood. After drying, the pellet, 80 µL of 1X TE buffer (10 mM Tris-HCL pH 8.0, 0.1 mM EDTA) was added into the microcentrifuge tube with the DNA pellet and stored at 4°C for at least 8 hr; this was then transferred and kept at -20°C.

Each DNA sample was quantified by using both a 1% agarose gel and Nanodrop spectrophotometer (ND 1000 V3.5.1) (Thermo Scientific, www.thermoscientific.com ). The Nanodrop spectrophotometer provides both the quality and quantity measurements based on the 280/260 ratio readings. However, this does not show fully the DNA quality, that is, if it is degraded or not. So, a 1% agarose gel with a λ DNA marker (22.2 ng/µL) was run at 60 volts for 20 min to further quantify the DNA. The agarose gels were visualized under the UV light using Genomic Solution software (Genomic Solutions, Harvard Bioscience, www.harvardbioscience.com ). After quantification, the DNA samples were diluted to 23 ng/µL concentration by adding 1 X TE buffer. These diluted DNA samples were then kept at -20°C until they were used, after which the samples were kept at -80°C as vouchers.

### AFLP process

A modified AFLP protocol ( Vos et al. 1995 ) was used to assess the genetic variability within and among *C. trifurcata* samples. DNA extracted from individual samples of *C. trifurcata* was used. The AFLP procedure consists of three basic steps: 1) DNA template preparation; 2) DNA template preamplification; and 3) selective amplification of the pre-amplified product. The DNA extracts were digested with EcoR1 and Mse1 restriction enzymes and ligated with specific adapters ( Table 2 ). The ligation product was diluted 1:10 with 1X TE buffer. This was then used as a template for the preamplification and selective amplification. Three combinations of two IRD-labeled ECOR1 primers (ACA and AAC) and two unlabelled MSe1 primers (CAA and CAG) (LI-COR, www.licor.com ) were used in this study to determine the genetic variability within and between the *C. trifurcata* samples ( Table 3 ). A control (all the AFLP reagents except DNA) was also run with the insect samples. AFLP products were separated in 6.5% denaturing polyacrilamide gels (LI- COR) and visualized in a Li-COR Gene Read IR 4200 DNA sequencer (LI-COR) for 2.5 hr at 45°C and 1500 volts. First and last lanes of the gel were loaded with 1 µL of IRD-labeled 50-700 base pair size standard marker (LI- COR).

**Table 2.**
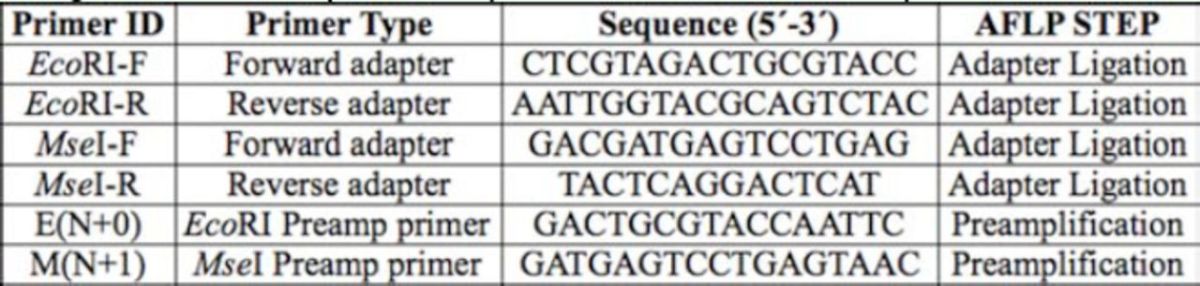
The sequences of oligonucleotide adapters, and primers used for AFLP analysis of *Cerotoma trifurcata* .

**Table 3.**
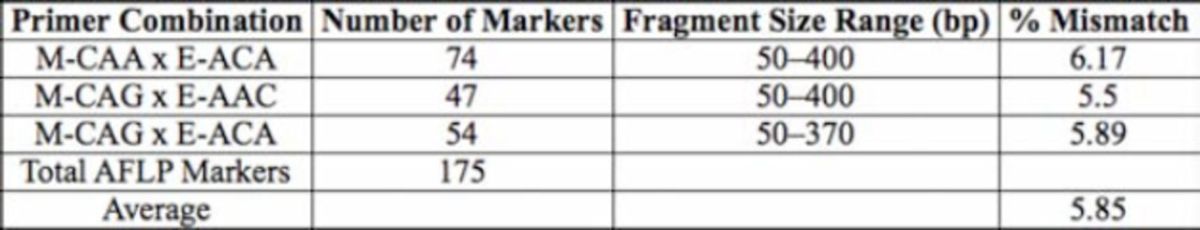
Selective AFLP primer combinations, number of markers, and fragment size for the primer pair combinations for *Cerotoma trifurcata* study.

### AFLP gel scoring

The AFLP bands were scored, using IRD-700 labeled 50-700 bp marker as a size reference with the SAGA Generation 2 software, version 3.2 (LI-COR). The visibility, sharpness, and repeatability of the bands were guiding criteria in marker selection for scoring. Profiles from multiple individuals were aligned and scored based on the presence (1) or absence (0) of a band on the AFLP gel, producing a binary data matrix.

DBOOD ( Coelho 2001 ) was used to evaluate the correlation between the coefficient of variation and the number of molecular markers observed, thus providing an estimate of the robustness of the data ( Hoelzel 1995 ). The binary data matrix was then used to estimate genetic similarity using the Jaccard index through the SIMQUAL procedure using NTSYSpc ( Rohlf 2000 ). Dendrograms were constructed to illustrate genetic similarity, following the methodology described by Sneath and Sokal (1973) . Bootstrap analysis was used (10,000 resamples), using BOOD-P software version 3.1 ( Coelho 2001 ) as a way of testing the reliability of the dataset for further analysis. The software package Arlequin version 3.1 ( Excoffier et al. 2005 ) was used to conduct the analysis of molecular variance (AMOVA). The AMOVA tests for genetic structure and genetic variability among groups, within groups, and within locations ( Schneider et al. 2000 ). ARLEQUIN (version 3.1) was also used for pairwise comparisons to test genetic divergence (FST - Wright’s inbreeding coefficient; Slatkin 1995 ). Genetic isolation was tested, comparing geographic distance and genetic dissimilarity, using the Mantel test ( Mantel 1967 ; Smouse et al. 1986 ) with 1000 permutations using ARLEQUIN version 3.1. POPGENE version 1.32 ( Yeh and Boyle 1997 ) was used to determine the degree of polymorphisms both within and between samples of *C. trifurcata* . Genetic differentiation between samples was assessed using Nei’s gene diversity index (GST) in POPGENE; gene flow was estimated from GST, expressed as (Nm) = 0.5 (1- GST)/GST ( McDermott and McDonald 1993 , Bonin et al. 2007 , Ryman and Leimar 2009 ). West (Nebraska) samples from 2008 and 2010 and East (Illinois/Ohio) samples from 2003 and 2010 were used to test for temporal genetic variation of *C. trifurcata* using total genetic diversity (HT), the Mantel test, and gene flow. Fall armyworm larvae, *Spodoptera frugiperda* (J.E. Smith) (Lepidoptera: Noctuidae) were used as the outlier group to test the robustness of the analytical tool in the POPGENE analysis.

### The effectiveness of AFLP markers

Each primer combination was used with 24 randomly selected individuals from the samples for assessing the genotyping error. Each primer combination was replicated three times, with these same 24 individuals, and the AFLP processes as initially done for the main experiment. The individual loci were then examined for mismatches among the three replicates. The error rate was calculated as the ratio of the total number of mismatches (presence or absence of a band) at a particular locus to the number of the replicated individuals ( Pompanon et al. 2005 ; Bonin et al. 2007 ). Loci with an error rate greater than 0.1 were rejected and not used in the study.

## Results

### The polymorphism and robustness of AFLP primers analyzed

A total of 175 loci ranging in size from 50 to 400 bp were observed using the three primer combinations ( Table 3 ). About 96.5% of the genetic variability was accounted for, indicating that there was sufficient number of markers for further robust analysis ( Coelho 2001 ).

The number of loci that were polymorphic for all the samples examined ranged between 124 and 168, with an average of 143 loci. The average loci polymorphism was 82% and ranged from 70.86% to 96% for Ames (Iowa) and Concord (Nebraska), respectively ( Table 4 ).

**Table 4.**
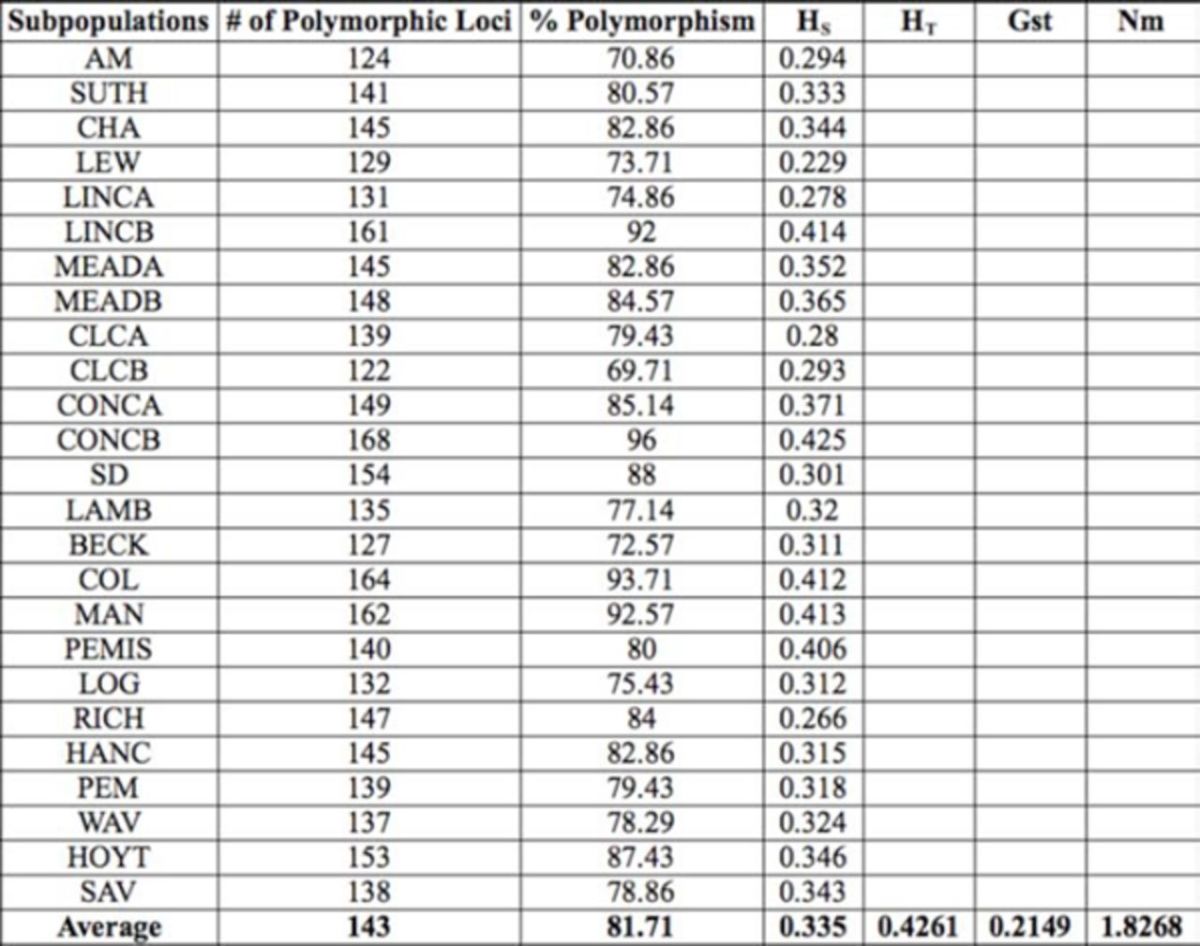
Genetic diversity estimates for all *Cerotoma trifurcata* subpopulations : number of polymorphic loci, percent loci polymorphism, heterozygosity (genetic variation) for a subpopulation (HS), heterozygosity for all *C. trifurcata* subpopulations (HT), gene flow among all subpopulations (Nm) and genetic variation between subpopulations (Gst).

### Temporal variation of *C. trifurcata* for Nebraska and Ohio samples

The total genetic diversity (HT), when comparing the 2008 and 2010 Nebraska samples, was 0.400, 0.410, 0.440, and 0.296 for Lincoln, Mead, Concord, and Clay Center, respectively. The proportion of total diversity among these samples (GST) was 0.136, 0.124, 0.089, and 0.032 for Lincoln, Mead, Concord, and Clay Center, respectively. Gene flow (Nm) for Lincoln, Mead, Concord, and Clay Center were 3.189, 3.527, 5.109, and 5.250, respectively, which indicates a high gene flow between the 2008 and 2010 samples. The total genetic diversity of the East samples taken in 2003 and 2010 was 0.385. The proportion of total diversity among samples was 0.175, and gene flow was found to be 2.362 for East samples. Mantel tests revealed no correlation between genetic and geographic distance for the western Midwest (Nebraska) region samples (r = 0.217; *P* = 0.181); the regression supported this finding (F1,26 = 3.07; *P* = 0.079; R2 =0.003). Similarly, results from the eastern Midwest (Illinois/Ohio) also showed no significant dependency of genetic distance on geographic distance (F1,26 = 2.86; *P* = 0.103; R2 = 0.099). Therefore, these samples (2003 and 2008) were used as part of the overall analyses.

### Genetic diversity, GST values, and gene flow

The average heterozygosity (HS) for individual samples was 0.335; the highest HS (0.425) was recorded at Concord, NE, and the lowest (0.229) was recorded at Lewis, IA ( Table 4 ). The average total heterozygosity (HT) for all the *C. trifurcata* samples (0.426) was high. This indicates that the overall heterozygosity of the *C. trifurcata* population is higher than that seen in individual samples. Analysis of multiple locations in POPGENE (Nei 1973; McDermott and McDonald 1993 ) for genetic diversity estimate (GST) for all the samples revealed a moderate level of differentiation (GST = 0.215) ( Table 4 ). The average gene flow (Nm) among the *C. trifurcata* samples was >1 (1.83) ( Table 4 ).

### Comparing genetic distance and geographical distance

**Figure 2.**
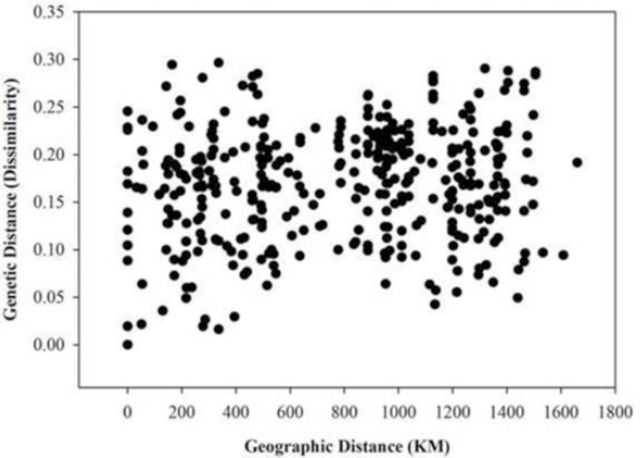
Genetic (dissimilarity) and geographic distance correlation among the *Cerotoma trifurcata* subpopulations sampled from western-Midwest USA (Nebraska), southern-Midwest USA (Missouri and Kansas), northern-Midwest USA (Minnesota and South Dakota), central-Midwest USA (Iowa), and eastern-Midwest USA (Ohio and Illinois). High quality figures are available online.

The results of the Mantel test for the entire data set revealed no significant correlation between geographic and genetic distance (r = 0.077, *P* = 0.180) in the *C. trifurcata* samples. This implied that there was no structure in the genetic variation among the samples in relation to either an increase or decrease in geographical distance. The majority of the dissimilarity matrixes had values between 0.1 and 0.25 ( Figure 2 ). In addition, the analysis of dissimilarity by geographic distance scatter plots revealed random dispersion of the matrices for all *C. trifurcata* samples ( Figure 2 ).

### Analysis of molecular variance

**Figure 3.**
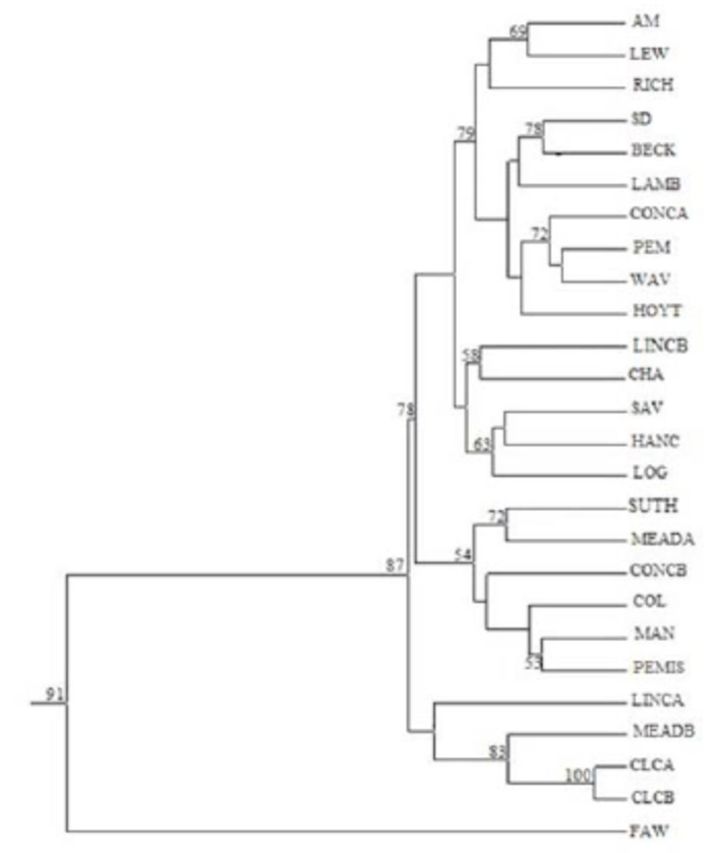
Dendogram illustrating the relationships among all the 25 *Cerotoma trifurcata* subpopulations collected from the Midwest USA, and an out group population of fall armyworm (FAW) collected from Iowa. Bootstrap values, when > 50%, are given at each of the forks (1000 replicates). High quality figures are available online.

Results from the AMOVA revealed the highest percentage of the variation (81.8%) from the total variation was from within *C. trifurcata* sample locations; 12.6% of the total genetic variation was from among samples within groups; and the variability among the groups accounted for 5.60% of the total *C. trifurcata* samples’ variation ( Table 5 ). The genetic divergence (0.182), which was measured by the fixation index (FST) as calculated by Arlequin (Excoffier et al. 1992), showed a moderate degree of genetic differentiation among *C. trifurcata* samples. The dendrogram clustering for the entire *C. trifurcata* samples of all locations did not show any distinctly defined groups or clustering that could separate the samples of *C. trifurcata* into geographic locations as defined for the Midwest USA in this study ( Figure 3 ). Moreover, the *S. frugiperda* larvae samples used as the outlier group to test the robustness of the analytical tool clearly separated from the *C. trifurcata* samples, indicating the reliability of the cluster analysis ( Figure 3 ).

**Table 5.**
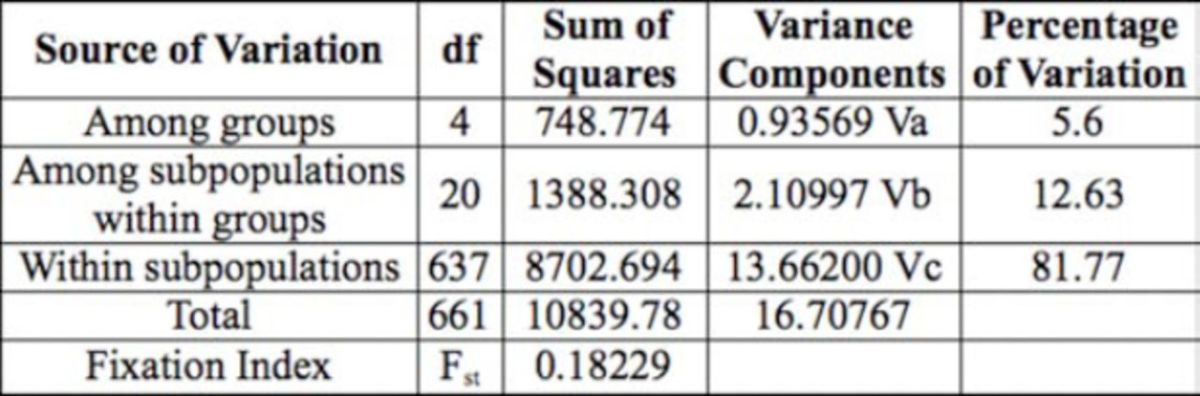
Analysis of molecular variance (AMOVA) for 25 subpopulations of *Cerotoma trifurcata* collected from five different regions of the Midwestern USA (North, South, East, West, and Central).

### The AFLP markers reproducibility

The reproducibility of the results by the three primer pairs, tested on 24 samples randomly selected from various samples, indicated that the AFLP approach was accurate and reliable. The average error rate found in this study was 5.85% ( Table 3 ). The maximum error rate acceptable varies with the scope of the study, but in most cases it should never exceed 10% ( Bonin et al. 2007 ). This implies that the error rate for this study was within the acceptable range; hence, the findings have at least 90% repeatability. Pompanon et al. (2005) emphasized that estimation of error rate should be a priority in AFLP studies. This study has revealed that AFLP is reproducible with an error rate of 5.85%. It is difficult to eradicate the genotyping errors because molecular assays and sample handling manually are not 100% ( Bonin et al. 2004 ).

## Discussion

The movement and dispersal of most organisms in the field, including *C. trifurcata* , is difficult to directly observe. The introduction of and advances in molecular biology provide a platform to explore this area by using genetic markers to provide the information needed to infer gene flow and migration tendencies of individuals among different sample locations. Analysis of *C. trifurcata* genetic structure is a key aspect to understand its population dynamics in agricultural scenarios. The development of effective pest management strategies relies on a multidisciplinary approach, and one component of this is knowledge of the population genetic structure of this pest. The formulation of pest management strategies depends on information such as population size, the factors affecting it, and even on the level of interaction among individuals from different locations, which all can influence the scale at which control practices should be implemented. This latter aspect can be addressed by an analysis and understanding of population genetic structure. The presence of geographic races or biotypes between or within geographic samples may indicate that different control approaches will be required ( De Leon et al. 2004 ).

The temporal variation results revealed relatively low genetic differentiation with good gene flow for *C. trifurcata* samples from both the West (Nebraska) and East (Illinois/Ohio) relative to year of sampling. There was also high gene flow (Nm) between the 2008 and 2010 samples for Nebraska as well as for the 2003 and 2010 Illinois/Ohio *C. trifurcata* samples. This relatively low GST and high Nm strongly supports that the majority of genetic variation is found within *C. trifurcata* samples rather than between years, prompting our decision to analyze all data together. Temporal population variation analyses provide a way of measuring realtime migration regardless of population history and identifying individuals in a sample as probable immigrants ( Paetkau et al. 2004 ). They also provide the most robust estimates possible of effective population size and migration rate ( Wang and Whitlock 2003 ).

The majority of the variation observed in this study was from within samples, followed by the variation among samples within the five regions , that is, southern Midwest, northern Midwest, western Midwest, eastern Midwest, and central Midwest, USA. The implication is that there is an adequate level of interbreeding between sample locations; hence, genetic variation has not led to a high level of differentiation among sample locations. Krumm (2005) , Clark (2005) , and Kondidie (2010) also showed similar observations of high within-population variation (as defined by AMOVA) in European corn borer and fall armyworm, respectively. The AMOVA results also indicated a low amount of genetic variation among groups, indicating that samples have not differentiated into separate genetic pools; this is further supported by the categorization of samples into the dendrogram. Neither between nor within the regions are isolated by any obvious geographic barriers that could lead to genetic fragmentation.

GST is the relative measure of genetic differentiation between samples. It is another indicator as to whether the majority of the variation is within or between the samples of interest. When GST is < 0.5, the implication is that the majority of variation is found within samples ( Clark 2005 ; Krumm et al. 2007), that is, individuals within samples are likely to be genetically different, but each sample contains the same complement of alleles in similar frequencies ( Clark et al. 2007 ). However, GST values >0.5, approaching 1, indicate that the majority of genetic variability is between samples. The GST for this study, 0.215, shows some genetic differentiation, but supports the notion that the majority of genetic variation was within the *C. trifurcata* samples, which further supports the AMOVA results.

The distribution of genetic variation within species is strongly linked to life-history traits, particularly dispersal and reproductive mode ( Hamrick and Godt 1990 ). This study revealed an overall Nm value among the *C. trifurcata* samples to be 1.826, indicating that there is reasonable gene flow among samples locations. Nm > 1 indicates the presence of sufficient gene flow between locations to counter actions leading to genetic differentiation and future genetic isolation ( McDermott and McDonald 1993 ). The level of gene flow observed in this study may be due to the fact that samples were in close proximity within the Midwest (yet there was over 1600 km east to west and 950 km north to south) with absence or limited impacts of spatial isolation. There is also a lack of geographic barriers that can hinder the *C. trifurcata* movement and dispersal. The average genetic diversity indicated a lack of genetic homozygosity. This implies more heterozygosity between individuals within samples and further supports the AMOVA results of high variation within a sample.

The Mantel test revealed no correlation of geographic and genetic distance for all the *C. trifurcata* samples. It is important to note that the relationship between genetic and geographic distances enables estimation of some demographic parameters such as effective population density and/or dispersal distance in the species of interest ( Rousset 2000 ). The insect population genetic structure depends on the capacity of a sufficient number of individuals crossing spatial and/or temporal barriers so that gene flow among the insects is not severely restricted. An UPGMA dendrogram from the cluster analysis constructed from the genetic distance matrix did not reveal distinct, conclusive separation of *C. trifurcata* samples into groups based on their geographic areas in relation to regions as defined in this study. Therefore, the samples are not genetically differentiated among the regions. The results further support that separate lineages of this pest have not emerged in the regions of this study.

The understanding of molecular data and the genetic principles can enhance the knowledge about insect origin, their diversity, and determination of their capabilities to reproduce in particular environments and communities. The study of insect genetic composition can also define a species in terms of their distinctive- ness, their relatedness, and their phylogenetic position. The lack of genetic differentiation observed in this study can, at least in part, be due to the expansion of soybean acreage, which provides continuous habitat and food resources for *C. trifurcata* . This ultimately leads to high possibilities of gene flow, resulting in the lowering of genetic differentiation. The movement of *C. trifurcata* has been associated with host (soybean) quality and availability ( Krell et al. 2003 ). The beetles tend to move to later planted soybeans, which will still be green later into the season, when early-planted soybeans begin to senesce ( Pedigo and Zeiss 1996 ); this is likely to spark the movement and spread of the beetles throughout the Midwest. Newsom and Herzog (1977) found that there is no or little movement by the overwintering *C. trifurcata* from an early-planted soybean area until the first generation was produced. Therefore, the majority of migration by these beetles is likely to occur after the first generation. A proportion of the population is capable of having seasonal migratory flights that spread the population when their original location gets negatively affected by adverse environmental conditions ( Coats et al. 1986 ). By so doing, the population gene pool will also be spread out and gene flow occurs. Insects’ capacity for movement, such as through flight, is in part affecting gene flow; hence, understanding the genetic relatedness of individuals is helpful for understanding their potential contribution to gene flow through natural movements.

There have been reports of *C. trifurcata* having short duration, unsustainable flights that only cover short distances ( Krell et al. 2003 ). However, this study indicates that these trivial movements may contribute to distributing the insect. It is of great interest to see how the increase in the soybean acreage and production, which leads to increases in food for these beetles, will shape the dynamics of *C. trifurcata* samples, especially their genetic structure. It is possible that this increase in food source may lead to the reduction in natural movements of *C. trifurcata* searching for soybean host plants, hence the increase in the residency time in a location and thus creating a possibility of higher genetic differentiation.

This is one of the first studies on the *C. trifurcata* molecular genetics. Based on these findings, it is concluded that there is no substantial genetic difference among the *C. trifurcata* sample locations in the Midwest, US. The findings of genetic variability of *C. trifurcata* support the conclusion that in the Midwest, this insect is genetically heterogeneous and part of a large, interbreeding population. Future research should extend the geographic range of samples to include other states outside the Midwest and even include samples from Canada. This will create a better understanding of this insect, which is growing in importance as an agricultural pest.

## Abbreviations

AFLP: amplified fragment length polymorphism
AMOVA: analysis of molecular variance
BLB: bean leaf beetle
